# Glycosaminoglycan-based biomaterials for growth factor and cytokine delivery: Making the right choices

**DOI:** 10.1016/j.jconrel.2019.10.018

**Published:** 2019-11-10

**Authors:** Daniel Hachim, Thomas E. Whittaker, Hyemin Kim, Molly M. Stevens

**Affiliations:** a Department of Materials, Imperial College London, London, SW7 2AZ, United Kingdom; b Department of Bioengineering, Imperial College London, London, SW7 2AZ, United Kingdom; c Institute of Biomedical Engineering, Imperial College London, London, SW7 2AZ, United Kingdom

**Keywords:** Glycosaminoglycan, Growth factors, Cytokines, Controlled delivery, Biomaterials

## Abstract

Controlled, localized drug delivery is a long-standing goal of medical research, realization of which could reduce the harmful side-effects of drugs and allow more effective treatment of wounds, cancers, organ damage and other diseases. This is particularly the case for protein “drugs” and other therapeutic biological cargoes, which can be challenging to deliver effectively by conventional systemic administration. However, developing biocompatible materials that can sequester large quantities of protein and release them in a sustained and controlled manner has proven challenging. Glycosaminoglycans (GAGs) represent a promising class of bio-derived materials that possess these key properties and can additionally potentially enhance the biological effects of the delivered protein. They are a diverse group of linear polysaccharides with varied functionalities and suitabilities for different cargoes. However, most investigations so far have focused on a relatively small subset of GAGs – particularly heparin, a readily available, promiscuously-binding GAG. There is emerging evidence that for many applications other GAGs are in fact more suitable for regulated and sustained delivery. In this review, we aim to illuminate the beneficial properties of various GAGs with reference to specific protein cargoes, and to provide guidelines for informed choice of GAGs for therapeutic applications.

## Graphical abstract

**Figure:**
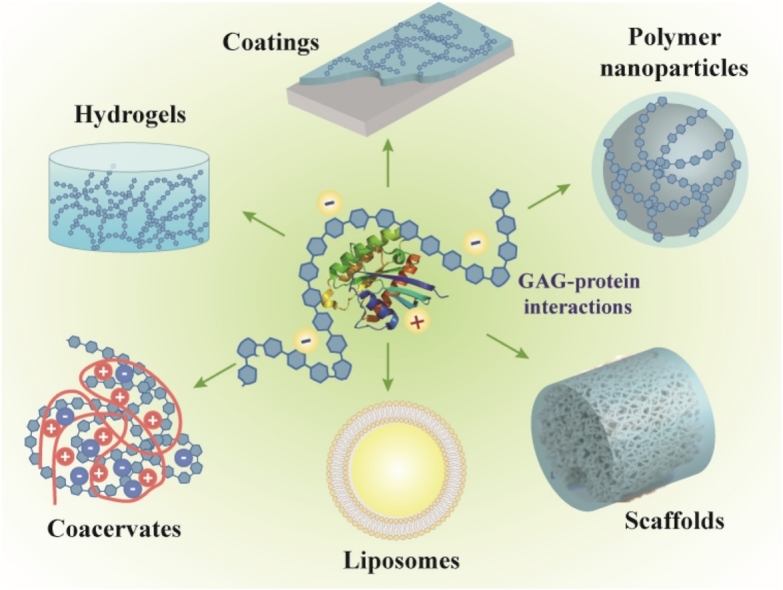

## 1 Introduction

**Fig. 1:**
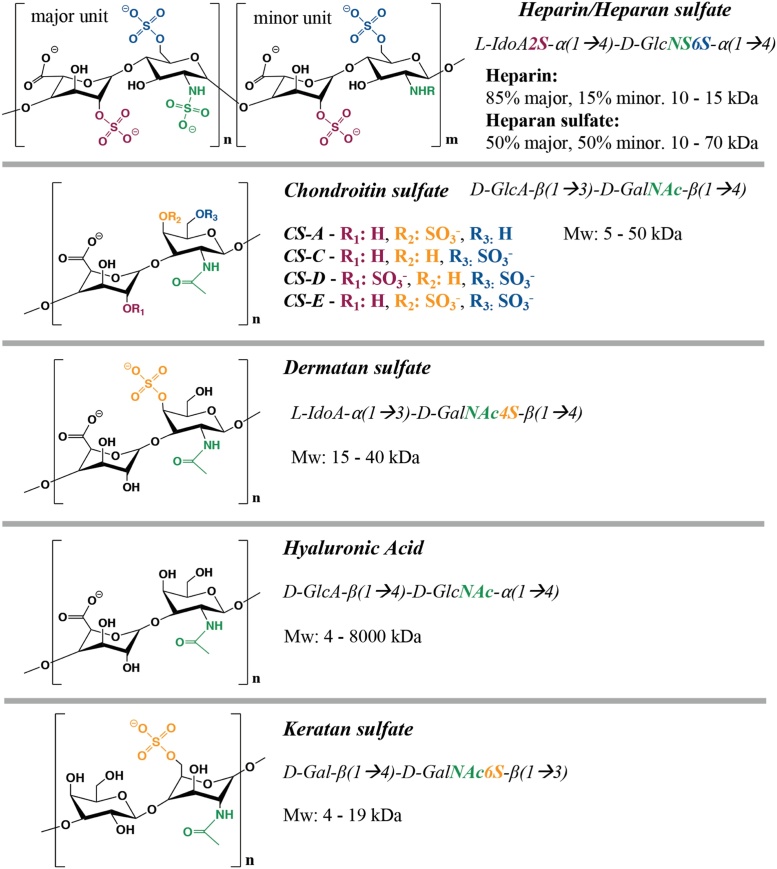
Common glycosaminoglycans found in animal tissues.

Glycosaminoglycan-based biomaterials have emerged as attractive candidates for drug delivery and tissue engineering applications. Glycosaminoglycans (GAGs) are naturally-derived polysaccharides comprising distinct sequences of disaccharide units, which are themselves typically composed of a combination of iduronic acid, glucuronic acid, glucosamine, galactose or galactosamine monosaccharides [[1], [2], [3]]. GAGs are often further modified by sulfation, the extent of which is heterogenous along the polysaccharide chains, forming regions with high and low charge density. Animal tissues contain multiple sulfated glycosaminoglycans such as heparan sulfate (HS), heparin, chondroitin sulfate (CS), dermatan sulfate (DS, also known as chondroitin sulfate-B) and keratan sulfate (KS), which can be distinguished by their sugar constituents and sulfation pattern. Some GAGs such as hyaluronic acid (HA) are not sulfated [2,4,5]. A scheme of the chemical structures of these GAGs can be found in Fig. 1.

GAGs are highly polar and interact strongly with water molecules [2,3,6,7], and act to maintain osmotic pressure and hence provide mechanical support within tissues. For example, HA, a major component in synovial tissue, has unique viscoelastic properties which act to reduce shear stress [8,9], making HA a promising biomaterial for joint tissue engineering applications. With the notable exception of HA, GAGs are mostly bound to proteins through O-linkages, while KS type I is the only GAG to be N-linked to core proteins. These are obtained by the sequential, semi-stochastic action of glycosyltransferase enzymes in the absence of a template, forming proteoglycans that contribute to regulation of cell signaling and function [1,3,4]. More recently, it has been shown that GAGs can bind to and sequester other proteins, such as growth factors and cytokines, to regulate their activity by either acting as a co-factor or by limiting their bioavailability [1,2,7,[10], [11], [12], [13], [14]]. In general, the biological activity and binding affinity of GAGs is dictated by their sulfation pattern, disaccharide unit sequence and 3D conformation; however, GAGs are also capable of unspecific binding of other positively charged proteins due to the negative charge provided by their numerous sulfate and carboxylic acid groups [1,2,10]. However, many proteins also contain specific evolutionarily conserved GAG-binding domains that mediate specific protein-GAG interactions. These domains often contain basic amino acids with small polar side chains (*i.e.* lysine and arginine), allowing flexibility and minimal steric hindrance for interaction with GAGs [1,15,16]. These more specific interactions are not purely electrostatic and are contributed to by hydrogen bonding, Van der Waals forces and hydrophobic interactions that are dependent on specific sequences and conformations of the GAG chains [1,17,18]. For example, ionic interactions were found to account for 30% of the binding between heparin and fibroblast growth factor 2 (FGF-2) [17], while the interaction of heparin and brain natriuretic peptide (BNP) was 94% attributable to hydrogen bonding and only 6% to ionic interactions [18]. Like all biomolecules, GAGs are dynamically synthesized and degraded, and as such are enzymatically degraded in a regulated manner by hydrolases. Upon degradation, any sequestered proteins in the chain are released.

**Fig. 2:**
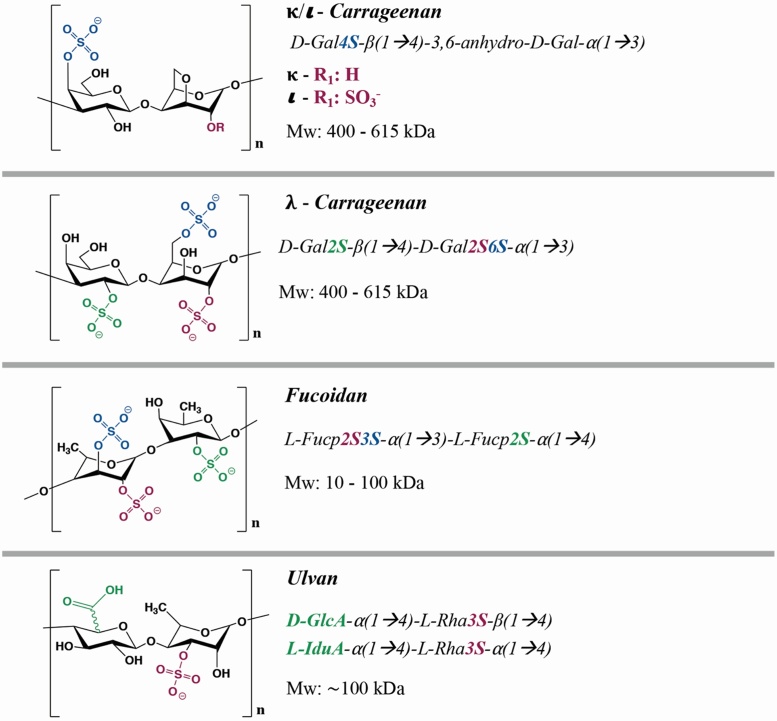
Common marine-derived sulfated glycosaminoglycans with medical potential.

Other sulfated polysaccharides that resemble GAGs have been identified in marine organisms. The most widely known marine GAGs in the medical field include fucoidan, carrageenan (from the galactan family) and ulvan, which are obtained from brown, red and green algae respectively [19,20]. Marine GAGs are typically composed of sulfated sugar units including iduronic acid, glucuronic acid, galactose, fucose and rhamnose (Fig. 2) [[19], [20], [21], [22]]. Much like classical GAGs, the biological activity, protein binding affinity and mechanical properties of marine GAGs depend on the sulfation pattern, sequence and conformation of their sugar units [19,21,23]. Although marine GAGs have so far not been extensively used in drug delivery and tissue engineering applications, their positive regenerative outcomes, low immunogenicity and abundance support their use as effective and sustainable alternatives to GAGs [19,[24], [25], [26], [27], [28], [29]].

Consequently, the properties of GAGs have attracted an increasingly high number of research groups to exploit them for delivery of proteins such as growth factors and cytokines [2,[30], [31], [32], [33]]. Regulated drug delivery to a therapeutic site is a long-standing clinical goal. Synthetic biomaterials in the form of hydrogels, nano- and microparticles, fibrous or sponge-like scaffolds and more have been developed and chemically modified or biofunctionalized to sequester and then release small molecule and protein cargoes [[34], [35], [36]]. Similarly to synthetic polymers, GAGs can be chemically modified to provide additional functionality. GAGs have been synthetically modified to enable delivery of a variety of bioactive molecules including small molecules, hydrophobic compounds and DNA, and to serve as major components of scaffolds for cell delivery and tissue constructs [2,[37], [38], [39]]. Chemical modification of GAGs can increase hydrophobicity or provide functional and crosslinking moieties (*e.g.* methacrylates) for scaffold fabrication. Carboxylic acid substitution on the uronic acid residues is the most widely used modification on GAGs, followed by modification of amino groups on non-acetylated/non-sulfated sugar residues. GAGs can be physically fabricated into different forms with varied mechanical properties, or alternatively can be present as a minor component within a bulk scaffold that provides desirable mechanical and structural properties. GAG-based biomaterials have been developed in a variety of physical forms for different applications, including hydrogels [24,[40], [41], [42], [43], [44], [45], [46], [47], [48], [49], [50], [51], [52], [53], [54], [55], [56], [57], [58]], surface coatings [[59], [60], [61]], nano- [[62], [63], [64]] and micro-particles [[65], [66], [67], [68]], coacervates [[69], [70], [71], [72], [73]], and fibrous scaffolds [[74], [75], [76], [77], [78]] **(**Table 1**)**. In many cases, chemical modification of GAGs assists synthesis or allows the addition of new properties of materials (*e.g.* addition of methacrylate groups to allow crosslinking with a synthetic PEG hydrogel [44]).

**Fig. 3:**
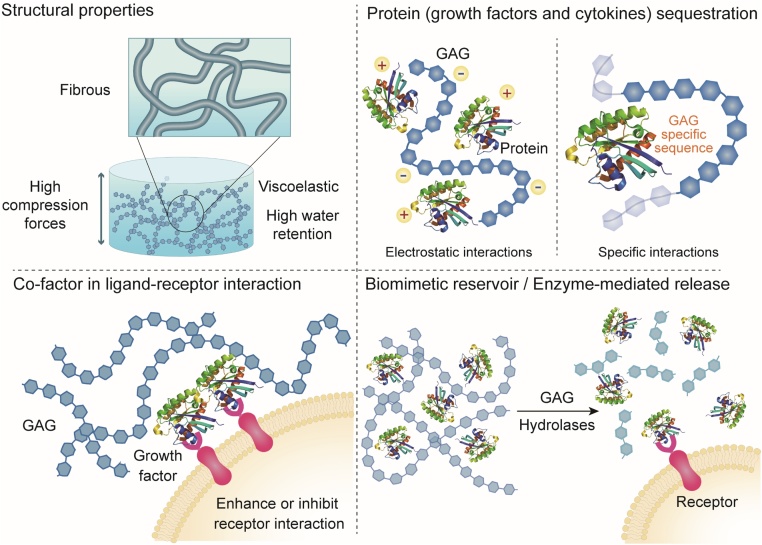
Properties of GAG-based biomaterials for protein delivery.

In contrast to synthetic materials, GAG-based biomaterials offer unique biological advantages when used for delivery of growth factors and cytokines (Fig. 3). Firstly, GAGs have a native ability to sequester and interact with these proteins, and some act as co-factors in the interactions between specific proteins and their receptors, often enhancing or facilitating bioactivity. For example, HS interacts with FGF-2 and FGF-2 Receptor 1 to enhance signaling [2,79]. Secondly, GAGs are naturally present in tissues and remodeled as part of normal healing processes; a GAG-based biomaterial therefore has the potential to release cargo to target cells in concert with naturally-regulated tissue remodeling processes - Heparanase expression can cause HS to release pro-inflammatory cytokines including interleukin (IL)-1β, IL-6, IL-8, IL-10, and tumor necrosis factor (TNF)-α in the extracellular matrix (ECM) [80]. Choice of GAGs led by these characteristics could augment the therapeutic effect of the cargo. However, a great proportion of research to date focuses on the use of a single GAG.

Heparin is by far the most commonly-used GAG for protein delivery. Widely used in the clinic as an anticoagulant, heparin is readily available in bulk and has a comparatively high degree of sulfation. As a result, it is a strongly negative polyelectrolyte and is able to effectively bind multiple growth factors and cytokines [30]. Heparin is a strong general candidate for a variety of applications; however, its ubiquity within the field may obscure the potential of other GAGs for the delivery of particular growth factors in specific applications. In this review, we will discuss the potential of alternative GAGs with reference to the prior use of heparin. We suggest that the selection of alternative GAGs can be guided by consideration of the following factors.

One factor to consider is whether the native biological function of the GAG is relevant to the application. A GAG may act as a co-factor to a loaded signaling protein to enhance signaling or may inhibit it by sequestration. It may also act independently of loaded cargo in a way that could beneficially affect the delivery site. Some GAGs have specific roles in the *in vivo* regulation of wound healing and other processes that might be exploitable in therapy. The GAG may also degrade in response to application-specific enzymatic activity, in a way that might be beneficial or obstructive to successful therapy.

A second factor to consider is the strength and specificity of the GAG-cargo interaction. Although many GAGs are relatively promiscuous and can bind multiple proteins *via* less specific bulk charge interactions, some proteins bind more strongly *via* evolutionarily conserved GAG-binding domains, which themselves can recognize specific saccharide sequences. A stronger binding can result in greater loading and slower release of cargo. Conversely, GAGs with promiscuous off-target binding (such as heparin) may cause adverse effects or inhibition of processes required for tissue homeostasis and repair [81].

Additionally, one can consider the potential chemical modifications that can be achieved with a given GAG – these may be required to add additional functionality, or to allow conjugation to a bulk material. However, these modifications can lead to changes in binding affinity for proteins depending on the functional group selected. This may impact specific GAG-protein interactions more severely than less specific bulk charge interactions. The anticoagulant activity of heparin has been reported to be reduced after carboxylate, amino and alcohol modification due to disruption of the specific interaction with Thrombin [2,42,[82], [83], [84]]. In general, the remaining unspecific affinity of GAGs for proteins after modification with these strategies is still sufficient for them to be useful as biomaterials for controlled delivery, as the percentage of modified disaccharide units does not typically exceed 40–50%. Modifications can themselves be used to improve loading. Sulfation patterns impact protein release and binding [[85], [86], [87]], and binding of charged cytokines to different GAGs was found to depend on charge spacing and density [88]. Synthetic desulfation and sulfation have been investigated as strategies to modulate the characteristics of GAGs [89] to allow tunable release of FGF-2 [85], platelet-derived growth factor (PDGF) [86], and vascular endothelial growth factor (VEGF) [87]. A review focused on the chemical structure of GAGs, binding affinity for proteins and delivery is available for further reading on this subject [2].

The immunological compatibility of GAGs also merits consideration. As native constituents of the human body, most GAGs have been found to have low immunogenicity compared to synthetic and even other naturally-derived biomaterials. Heparin, CS, and HA have been shown to prevent recruitment and adhesion of leukocytes *in vitro* and when used in coatings for implanted biomaterials [90]. These GAGs also promote anti-inflammatory effects, the mechanisms of which are currently not fully understood [[91], [92], [93]]. While Fucoidan and other marine-derived GAGs also appear to be immunologically innocuous or beneficial in terms of the innate immune response [94,95], there exists more potential for an adaptive response due to its xenogeneic origin, the risk of which is as yet unclear. This risk is increased by the possibility of contamination with allergens and other xenogeneic factors from the source organism during production. Appropriate GMP-compliant purification protocols exist for GAGs in common clinical use but may require adaptation for more novel GAGs. Less commonly-used GAGs may not be commercially available at sufficient purity for representative *in vivo* testing.

The final consideration is cost and availability. Heparin is readily available due to the vast industry supporting its production as an anticoagulant for medical care, which extracts heparin from the gastric mucosa of an estimated 10^9^ pigs annually [96]. HA and some forms of CS are available at similar costs, but most other GAGs are costlier and only available in smaller quantities. This has discouraged their use – however, they may still be practical for use in smaller quantities as the biofunctional component of a hybrid material. These less commonly-used GAGs may have significant advantages over the more well-established ones.

The present review aims to serve as a guide for the identification of optimal combinations of GAG-based biomaterials and bioactive cargoes, based on evidence from and discussion of an extensive list of studies on GAG-based delivery systems and GAG-protein interactions. We will consider the factors underlying GAG choice in relation to commonly-used protein cargoes. For most applications, there is a history of prior use of heparin, which will be evaluated to allow comparison to alternative GAGs. We will also identify areas where informed choice based on our suggested considerations is challenging due to a lack of available data and suggest how these areas might be clarified.

## 2 GAG-mediated delivery of growth factors

In addition to the non-specific electrostatic interactions commonly present between negatively charged GAGs and positively charged (pI > 7) proteins, several growth factors present specific domains that have shown high binding affinity for heparin or heparan sulfate (heparin-binding domains). In the extracellular matrix (ECM) and on the cell surface, HS proteoglycans have a wide range of cell signaling functions including activation of fibroblast growth factor (FGF), transforming growth factor beta (TGF-β), Hedgehog and Wnt signaling pathways [97]. Heparin-based biomaterials therefore seem a logical choice for delivery of such growth factors. However, while this may be true for many growth factors, research has revealed that in some cases other GAGs may be more suitable; due either to a more effective interaction with the growth factor and receptor, more regulated degradation offering an opportunity for control or bio-responsiveness, or apparently greater importance in *in vivo* wound healing processes. This could allow greater mimicry of the biological activity and bioavailability of growth factors in the native target, leading to a more effective therapeutic response. A summary of GAG-based delivery systems for delivery of growth factors and cytokines is shown in Table 1.

**Table 1:** GAG-based delivery systems for delivery of growth factors and cytokines.

**Family**	**Protein**	**GAG**	**Delivery System**	**References**
Fibroblast growth factor (FGF)	FGF-1	Heparin	Coacervates	[70]
	FGF-2	Heparin	Hydrogel	[[40], [41], [42], [43]]
			Nanoparticles	[62]
			Micelles	[98]
			Coacervates	[[69], [70], [71],^99^]
			Porous scaffold	[28]
		CS	Hydrogel	[44]
		Fucoidan	Porous scaffold	[28]

Vascular endothelial growth factor (VEGF)		Heparin	Hydrogel	[45]
			Nanoparticles	[63]
			Coating	[[100], [101], [102]]
			Synthetic scaffold	[103]
			ECM Scaffold	[104]
		HA	Coating	[102]

Platelet-derived growth factor (PDGF)		Heparin	Microparticles	[105]
			Coating	[59,60]
			Scaffold	[106]
		CS	Scaffold	[107]

Heparin binding - epidermal growth factor (HB-EGF)		Heparin	Hydrogel	[46]
			Coacervates	[108,109]
		Sulfated HA	Hydrogel	[47]

Bone morphogenetic protein (BMP)	BMP-2	Heparin	Microparticles	[[65], [66], [67]]
			Hydrogel	[[48], [49], [50]]
			Scaffold	[[110], [111], [112], [113]]
		CS	Coating	[114]
			Scaffold	[[74], [75], [76]]
			Hydrogel	[52]
		DS	Hydrogel	[51]
		HA	Hydrogel	[[51], [52], [53]]
	BMP-4	CS	Hydrogel	[115]
			Coating	[116]

Neural growth factor (NGF)		Heparin	Scaffold	[78]
			Hydrogel	[55]
		CS	Hydrogel	[54,55]
		HA	Hydrogel	[55]

Sonic hedgehog (SHH)		Heparin	Coacervates	[72]

Brain-derived neurotrophic factor (BDNF)		CS	Hydrogel	[44]

Interleukins (IL)	IL-4	Heparin	Hydrogel	[117]
			Scaffold	[77]
		DS	Coating	[118]
	IL-6	Heparin	Coacervates	[73]
	IL-10	Heparin	Coacervates	[72]
		CS	Hydrogel	[44]
		HA	Hydrogel	[56]

Transforming growth factor β1 (TGF-β1)		CS	Microparticles	[68]
		Carrageenan	Hydrogel	[24]

Stromal cell-derived factor 1a (SDF-1a)		Heparin	Hydrogel	[57]
			Nanoparticles	[64]
		HA	Hydrogel	[58]
			Coating	[119]

Monocyte chemoattractant protein - 1 (MCP-1)		DS	Coating	[61]
		Heparin	Coacervates	[73]

Tumor necrosis factor a (TNF-a)		CS	Microparticles	[68]

Combination	FGF-2/VEGF	Heparin	Hydrogel	[41,120]
	VEGF/PDGF	Heparin	Scaffold	[121]
			Coacervates	[122]
		HA	Nanoparticles	[123]
	MCP-1/IL-4	DS	Coating	[61]
	SDF-1a/VEGF	Heparin	Nanoparticles	[64]
	IL-10/FGF-1	Heparin	Coacervates	[70]
	VEGF/MCP-1/IL-6	Heparin	Coacervates	[73]
	SDF-1a/BMP-2	HA	Hydrogel	[58]

### 2.1 Fibroblast growth factor (FGF) family

**Fig. 4:**
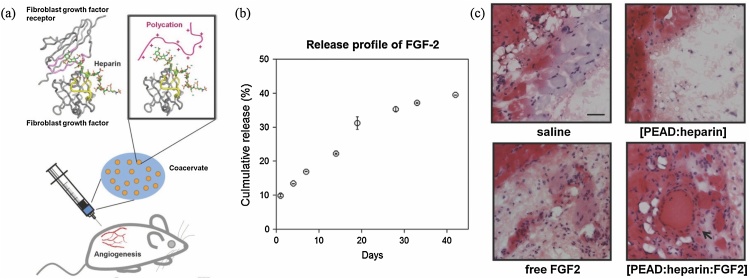
(a) Heparin-PEAD coacervates for sustained delivery of FGF-2. (b) Release profile of FGF-2 *in vitro*. (c) Hematoxylin and eosin staining of subcutaneous tissues after 4 weeks. For the saline, delivery vehicle, and free FGF-2 groups, there was no clear growth of vasculature in the subcutaneous region. Contrastingly, in the coacervate group, new blood vessels were observed with a closed inner layer of nucleated cells surrounded by smooth muscle bundles (arrow). Scale bar: 50 μm. Adapted from [69,99] (*in vitro* and *in vivo* studies respectively, on same coacervate formulation). Fig. 4b reprinted with permission from Elsevier.

The most commonly-used pro-angiogenic growth factors in drug delivery systems and tissue engineered constructs are Fibroblast Growth Factor - 2 (FGF-2), vascular endothelial growth factor (VEGF) and platelet-derived growth factor (PDGF). The interactions between heparan sulfate, heparin and the Fibroblast Growth Factor (FGF) family have been extensively studied and are well characterized, particularly those involving FGF-2 (also known as basic FGF, bFGF). This family comprises 22 members with mitogenic activity, many of which have been used in clinical settings to elicit tissue repair in mature tissues [79]. FGF-2 has been widely used to promote angiogenesis and cell proliferation in constructs for regenerative medicine. Binding of FGF to the ECM is known to be mediated by specific interactions with HS [124]. It was reported that a trisaccharide motif found in HS containing 2-O sulfated iduronic acid and 6-O desulfated glucosamine strongly binds to FGF-1, and a mono-O-sulfated HS-derived hexamer with a single 2-O sulfated iduronic acid binds to FGF-2 [125]. In addition, heparin strongly binds FGF-2 *via* a penta-saccharide sequence, 2-O sulfated iduronic acid and 6-O-desulfated glucosamine, forming a ternary complex that strongly enhances binding to FGF receptors, enhancing the cell response [2,79]. The established interactions of heparin and FGF-family proteins likely reflect *in vivo* functions of heparan sulfate, as heparin is only found in significant quantities in mast cell granules in humans [126,127], whereas heparan sulfate (which shares the same penta-saccharide sequence) is abundant in many tissues. However, HS is not commercially available in bulk and so heparin-mediated delivery of FGF2 has been widely used in numerous delivery systems including hydrogels [[40], [41], [42], [43]], nanoparticles [62], micelles [98], coacervates [[69], [70], [71]] and other scaffolds [28]. Heparin-based biomaterials have been shown to achieve loading efficiencies close to 100% and to protect FGF-2 from proteolytic degradation, providing sustained release over a period of weeks. Bioactivity is also preserved and often higher than that of free FGF-2, showing enhanced endothelial cell differentiation *in vitro* and improved angiogenesis *in vivo* (Fig. 4). As the sulfation pattern is most important in driving binding interactions with these growth factors, modifications on carboxylic acid and de-acetylated amino residues of these GAGs do not typically seriously impact the binding and loading efficiencies. For example, a 22% methacrylation degree of heparin *via* de-acetylated amino residues resulted in retention of 80% of the initial binding efficiency for FGF-2, compared to non-modified heparin [42].

Due to the presence of a specific, evolutionarily conserved interaction between FGF-family proteins and heparin/HS, heparin-based biomaterials are good candidates for delivery of these growth factors. Particular focus has been given to delivery of FGF-2. It is worth noting that some members of this family, including FGF-1, FGF-4 and FGF-18, bind heparin but by alternative binding domains – a result of convergent evolution [10,128,129]. Additionally, different FGFs may show distinct binding preferences or biological effects in presence of other GAGs, and hence GAGs other than heparin should be also be considered as potential carriers, depending on the application. Significant binding affinity for FGF-2 has also been observed in CS-E and DS [10,130,131], in which the 4-O sulfation of the N-acetyl galactosamine sugars seems to be key for interaction with both FGF-2 and FGF-7 [132]. A CS hydrogel scaffold showed a high binding affinity for FGF-2 and allowed self-renewal of neural stem cells, demonstrating potential for neural tissue repair [44]. In wound healing, DS is abundant and binds FGF 2, 7, and 10 to activate cell proliferation, which was abolished when using GAG preparations with a higher degree of sulfation [132]. In addition, DS exhibited stronger binding affinity for FGF-10 and enhanced migration of keratinocytes more effectively than heparan sulfate or other CSs found in wounds [133], suggesting that DS and FGF-10 may be a promising combination for delivery in wound healing. Fucoidan, a marine GAG derived from brown algae, has been modified to increase sulfation in order to increase binding to FGF-2, enabling sustained delivery of FGF-2 from a Fucoidan-chitosan-alginate scaffold to promote fibroblast migration *in vitro* [28]. When available information is not available regarding the binding affinity of particular members of the FGF family, preliminary binding studies could be performed to confirm the most suitable GAG, 4-O sulfated GAGs being the likeliest candidates.

### 2.2 Vascular endothelial growth factor (VEGF)

Heparin-based biomaterials have also been used for sustained delivery of VEGF, including hydrogels [45], nanoparticles [63], coatings [[100], [101], [102]], synthetic scaffolds [103] and decellularized scaffolds [104]. VEGF-A is a key regulator of angiogenesis, and alternative splicing of VEGF-A results in several isoforms with different binding affinities. All VEGF-A isoforms except VEGF_121_ have been found to interact with heparin and HS at least partially *via* a specific binding domain, rather than by bulk charge interactions [[134], [135], [136]]. VEGF_165_ is the most commonly-used isoform in medical research, and most studies use interactions between heparin and this isoform. Heparin/chitosan nanoparticles loaded with VEGF_165_ and immobilized in decellularized bovine jugular vein scaffolds provided sustained release for several weeks *in vitro*, and stimulated fibroblast infiltration, matrix deposition and vascularization after subcutaneous implantation *in vivo* [63]. Heparin-VEGF_165_ multilayered coatings have been fabricated on decellularized aortic heart valves, which provided both hemocompatibility and release of VEGF_165_ over 5 days, stimulating migration, adhesion and proliferation of endothelial progenitors within the scaffold [100]. Similarly, coating a hydroxyapatite scaffold with VEGF_165_-containing heparin-collagen multilayers improved the retention and proliferation of MSCs, leading to increased vessel formation after 28 days post-implantation [101]. Heparinized polycaprolactone (PCL) scaffolds effectively retained VEGF_165_ and promoted the proliferation and differentiation of MSCs into endothelial progenitor cells [103]. Demineralized bone crosslinked with heparin showed increased loading efficiency and a release of 80.6% of VEGF_165_ after 3 days, and improved angiogenesis after subcutaneous implantation [104].

Most studies on GAG-mediated VEGF loading and release have focused on heparin – however, VEGF-loaded HA has been used as a coating on allogeneic bone-patellar tendon-bone implants. This provided a steady state release of VEGF over approximately 40 h and led to improvements in revascularization of the implant in a rabbit model of ligament reconstruction [102]. HA is potentially advantageous in this application as it is itself able to stimulate angiogenesis independently of VEGF, by binding to and activating the Receptor for HA-Mediated cell Motility (RHAMM) [137]. However, these and other binding studies of VEGF and glycosaminoglycans have shown that the binding affinity of VEGF for HA is weaker than for other sulfated glycosaminoglycans. The strongest interaction was found to be with heparin/HS [138]. These findings suggest that the interaction between VEGF and HA occurs mainly *via* ionic interactions with carboxylic acid groups rather than interactions with sulfate groups, with a contribution from non-electrostatic interactions, as also suggested for other proteins [2,139].

### 2.3 Platelet-derived growth factor (PDGF)

Heparin substantially increases the signaling potency of FGF-2 by acting as a co-factor – however, binding between certain GAGs and growth factors can cause inhibition of growth factor signaling, by sequestration in an inactive complex. The sequestered growth factors may then be gradually released by slow unbinding or enzymatic degradation, leading to sustained, regulated activity over time. These properties make GAGs suitable biomaterials for controlled long-term delivery of these factors *in vivo.* However, it is worth noting that when GAGs are not pre-loaded with a growth factor, they may sequester native growth factors from tissue. The mitogenic and chemotactic activity of PDGF has been shown to be downregulated in the presence of heparin or CS [140,141] by an indirect mechanism consistent with sequestration. However, when GAG-based biomaterials containing PDGF are used for delivery, they are pre-loaded with protein and so there is no binding availability to enable sequestration of PDGF or other factors from the native tissue. Delivery of PDGF using GAGs has been achieved, and it has been confirmed that the observed effects are due to the delivered PDGF. For example, heparin-decorated microparticles developed for long-term release of PDGF exerted an anti-inflammatory activity in a rabbit model of tendinitis [105]. Electrochemically aligned and heparinized collagen sutures loaded with PDGF were capable of providing release for up to 15 days, resulting in improved biomechanics and vascularization during post-laceration repair of chicken flexor tendons 12 weeks post-surgery [59,60]. Similarly, a heparin/fibrin-based scaffold to deliver PDGF for 10 days on a canine flexor tendon model increased cell activity and functionality, but not the structural properties of the sutured tendon [106]. CS was also used to facilitate the delivery of PDGF for guided bone regeneration. A porous CS-chitosan sponge was fabricated by a lyophilization method, and PDGF was incorporated into the sponge. PDGF was released in a sustained manner, significantly improving osteoblast proliferation compared to controls [107].

### 2.4 Heparin-binding epidermal growth factor (HB-EGF)

HB-EGF is a member of the EGF family that is critical to wound healing and contains a conserved heparin-binding domain involved in regulating its localization *via* interactions with extracellular GAGs [[142], [143], [144], [145]]. GAG-based biomaterials have therefore been explored to deliver HB-EGF, as they could be used to mimic this aspect of the wound healing process. Heparin-based hydrogels loaded with HB-EGF exhibited first order kinetics with sustained release of 90% of their content during 2 weeks, inducing the proliferation of human corneal epithelial cells [46]. Heparin-based coacervates loaded with HB-EGF provided sustained and steady release over 10 days *in vitro* and enhanced the migratory and proliferative activity of keratinocytes [108]. *In vivo*, heparin-based coacervates led to an accelerated wound re-epithelization in both normal and diabetic mice [108,109]. HB-EGF also interacts with other GAGs. The driving forces of these interactions are likely predominantly electrostatic interactions between positively charged lysine/arginine-rich clusters in the heparin-binding domain and the sulfate residues in the GAGs, as suggested in a comparative study between HA and sulfated HA [47]. Both relatively specific interactions (*i.e.* with heparin) and unspecific interactions are potentially useful for protein loading and release. Specific interactions have some advantages, but an advantage of unspecific interactions is that they are more amenable to engineering, as illustrated in this study. Chemically-modified derivatives of HA with increased sulfation had 10% greater binding affinity for HB-EGF compared to native HA, leading to a more extended release of growth factor and improved promotion of epithelial growth [47]. In general, sulfation is a viable strategy to improve the affinity of non-specific GAG-protein interactions. Other GAGs may have as yet unestablished specific interactions with EGF-family proteins; studies regarding the effects of EGF on neural development in the presence of CS have revealed an important regulatory role of this GAG in EGF-mediated effects on cardiac development and neurogenesis [145,146]. EGF and CS are therefore likely to interact, although this binding has not yet been tested in delivery systems.

### 2.5 Pro-angiogenic combination therapy

As angiogenesis *in vivo* is stimulated by the orchestrated action of FGF-2, VEGF and PDGF amongst other factors, some recent biomaterial design has focused on sequential delivery of multiple factors rather than single-factor regimens [41,122,[147], [148], [149]]. Heparin-based hydrogels have been used for coordinated delivery of both FGF-2 and VEGF, the effects of which were synergic on endothelial cell proliferation and vascularization compared to single delivery regimens [41,120]. Heparinized polyurethane scaffolds were made to sequentially deliver VEGF over an initial 24 -h period, followed by PDGF for more than 7 days [121]. Vessel ingrowth was found to be a consequence of VEGF delivery, while PDGF increased vascularization. This combined regimen significantly increased the formation of arterioles, a result not achieved by either factor in isolation. A novel delivery system consisting of heparin-poly(ethylene arginyl aspartate diglyceride) (PEAD) coacervates embedded into a fibrin gel was used to sequentially release VEGF and then PDGF in an animal model of myocardial infarction improved revascularization after infarction, reducing cardiomyocyte death and infarct size *in vivo*, leading to improved cardiac function [122].

In addition to the use of heparin, HA and chitosan nanoparticles have also been used to provide sequential delivery of VEGF and PDGF, with a loading efficiency of 94% and 54%, respectively. Similarly, while release of VEGF occurred completely during the first 24 h, the release of PDGF was maintained for a week [123]. This suggest that binding affinity of HA for PDGF is stronger than for VEGF, which in this case is a convenient strategy to provide sequential release. Alternatively, if detailed data were available on the binding kinetics of multiple GAGs and proteins, it might be possible to design a biomaterial containing a mixture of GAGs in order to achieve a particular timing and sequence of delivery for multiple proteins.

### 2.6 Bone morphogenetic proteins (BMP) and the transforming growth factor beta (TGF-β) superfamily

BMPs are growth factors within the TGF-β superfamily, and are known to have major signaling roles during the development of cartilage, heart and neural tissue, but are also known to participate in post-developmental signaling, particularly during postnatal bone formation [150]. BMP-2 and BMP-7 were amongst the first BMP family proteins to be discovered and have been extensively studied and approved for clinical use to induce cartilage and bone formation. Degradation and short span of these growth factors *in vivo* led to the use of high doses to promote a therapeutic effect in the clinic; however, this led to a high rate of adverse effects (*e.g.* ectopic bone formation and cervical spine swelling) [151]. Therefore, several delivery systems have been developed to protect these growth factors and provide sustained release, decreasing the administered dose to avoid damaging side-effects by sequestering the bulk of the drug. GAGs are known to bind BMPs, again *via* lysine/arginine-rich negatively-charged domains [152], and so are a candidate biomaterial for this application.

BMP has been delivered by heparin-based materials in many forms, including microparticles [[65], [66], [67]], hydrogels [48,49] and other scaffolds [[110], [111], [112], [113]]. Microparticles fabricated from heparin with a variety of sulfation patterns were studied to determine their effects on both the binding and release of BMP-2. While degradation of these particles was dependent on the heparin content, a higher loading efficiency was observed with higher degrees of sulfation. Quantification of BMP-2-stimulated alkaline phosphatase (ALP) activity in C2C12 cells revealed that the bioactivity of BMP-2 using highly sulfated heparin was approximately 4 times higher than BMP-2 combined with less-sulfated heparins or soluble BMP-2 [65]. Another study showed that heparin-based microparticles provided sustained release of BMP-2 over 28 days, with a total released dose of 20% and a low burst release of less than 10% during the first 6 h. BMP-2-loaded microparticles stimulated ALP activity in C2C12 myoblasts in culture, to a similar degree to soluble growth factor [66]. In another study, heparin-based microparticles loaded with BMP-2 were immersed in an alginate hydrogel to provide localized and sustained delivery of BMP-2 into a large femoral defect. This induced both ectopic and orthotopic bone formation after implantation; however, less bone formation was observed than for BMP-2 delivery *via* the hydrogel alone. The authors suggested that optimization of the release kinetics from heparin may be required to identify the most beneficial timescale for release [67].

An injectable hydrogel for sustained delivery of BMP-2 was made by combining a heparin-fibrin conjugate with thrombin, which was capable of releasing ≈ 90% of the growth factor over 13 days, compared to 3 days for normal fibrin gels. The released BMP-2 increased alkaline phosphatase activity of osteoblasts in culture, which was absent after stimulation with fibrin gels alone. Implanting BMP-2-loaded heparin gels into the hind limb muscle pockets of rats promoted the highest bone formation among all groups [48]. Photocrosslinked heparin-alginate hydrogels enabled sustained released of bioactive BMP-2 over 3 weeks without initial burst release. Implanted hydrogels induced 1.9-fold higher bone formation in the peri-implant zone and 1.3-fold higher calcium content than alginate hydrogels 8 weeks post-implantation. Similarly, this gel was also able to provide release of TGF-β1 over the course of 3 weeks [49].

Heparin-conjugated collagen sponges were made to incorporate high doses of BMP-2, which exhibited an initial burst followed by sustained release of BMP-2 for weeks [110]. Compared to non-heparinized sponges, the release of BMP-2 showed higher expression of bone markers, osteoclast activity and more uniform mineralization 7 days post-implantation in bone defects. Another study using collagen/fibrin sponges conjugated to heparin showed 80% release of the total loaded BMP-2 in 20 days, while non-conjugated sponges released the same amount in 6 days. Heparin-conjugated sponges enhanced osteogenic efficacy by increasing ALP activity *in vitro* and bone density in a calvarial defect model, using half of the dose used for non-heparinized sponges [111]. Bone scaffolds for sustained release of BMP-2 were made by crosslinking heparin-chitosan with TPP and incorporation of Si-CaP powder. Loading efficiency ranged from 59% to 75%, depending on the initial BMP-2 concentration. 50% of loaded protein was released after 14 days, and 90% after 45 days [112]. Granules made of collagen, tricalcium phosphate and hydroxyapatite were conjugated to heparin, which increased the loading efficiency of BMP-2 compared to non-coated scaffolds and provided sustained release up to 21 days [113].

While the majority of studies investigating GAG-mediated BMP delivery have focused on heparin, other GAGs such as HS and CS have also been used. The use of HS has been controversial. HS proteoglycans influence BMP signaling pathways by interacting with secreted BMP antagonists such as Chordin and Noggin [[153], [154], [155]], and so could inhibit BMP signaling. HS is thought to potentiate the Chordin-induced antagonism of BMP signaling by binding with Chordin and mediating its retention and uptake by cells [154]. On the other hand, results from comparative studies between CS and heparin for delivery of BMPs have shown that CS may be superior for the delivery of BMP-2 and BMP-4. GAG-based coatings synthesized by layer-by-layer deposition have been made using both native heparin, oxidized heparin, and CS as polyanions in combination with chitosan as a polycation. All coatings released BMP-2 over a period of 4 days; however, the heparin-based coatings released larger amounts of protein than those based on CS, while both oxidized GAGs released even less. This suggest higher affinity of oxidized GAGs for BMP-2 followed by CS and then heparin. Higher retention or affinity of BMP-2 was correlated with increased cell adhesion, proliferation and differentiation towards osteoblast, with oxidized CS presenting the highest levels [114]. Another study showed that, in particular, disulfated CS-E efficiently sequestered BMP-4 and enhanced osteoblast differentiation, cell growth, ALP activity, collagen synthesis and bone mineralization, while heparin was found to increase ALP activity and mineralization only [156].

CS has been used for sustained delivery of BMPs in the form of coatings [114] and scaffolds [[74], [75], [76]]. A CS-based scaffold was compared to collagen sponges (the standard clinical delivery method) as BMP-2 delivery systems. *In vitro* extended release of 7 μg of BMP-2 over 16 days was observed in the CS scaffold but not the collagen sponge, stimulating bone formation in a critically sized femoral defect in rats [74]. CS-collagen scaffolds were coated with poly-L-lactide (PLLA) and sucrose acetate isobutyrate to extend the release of BMP-2, resulting a release of 50% of growth factor over 15 days. Histological examination revealed a dose-dependent periosteal ossification of the scaffold [75]. A porous CS-chitosan scaffold was further modified with an apatite coating for delivery of BMP-2 and enhanced cellular response. The apatite coating reduced the initial burst release and sustained release was provided over 5 days. In a mandibular defect model, BMP-2 enhanced bone matrix formation 8 weeks post-implantation [76].

**Fig. 5:**
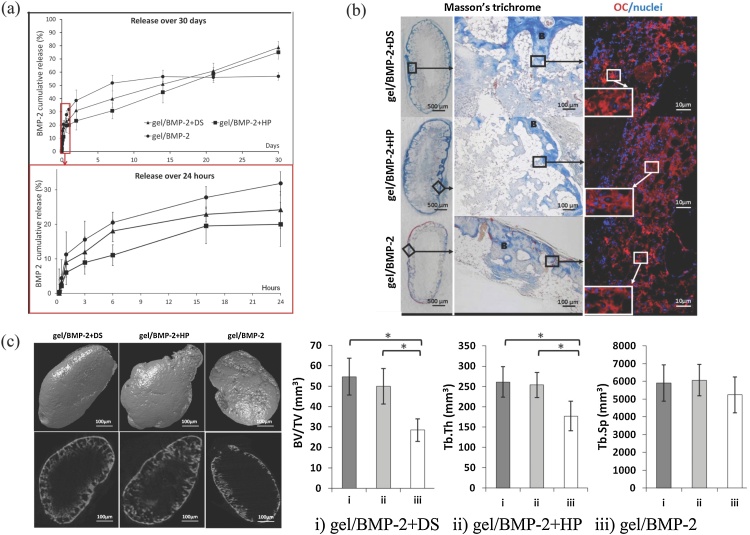
(a) BMP-2 cumulative release from HA hydrogels containing DS, heparin and control. (b) Ectopic bone formation 6 weeks post-implantation, evaluated histologically *via* Masson’s Trichrome staining and osteocalcin (OC) immunostaining. B indicates a trabecular bone structure. Nuclei were stained with DAPI. (c) Micro-CT analysis. 3D (upper row) and 2D (lower row) images of the surface of ectopic bone formed 6 weeks post-injection. The average bone volume/tissue volume (BV/TV) ratio, the average trabecular thickness (Tb.Th.) and the average trabecular separation (Tb. Sp.) were calculated. Values are the mean ± SD for n = 6; * p < 0.05. Adapted from [51].

Comparative studies were also performed between heparin and DS. HA-hydrogels were precomplexed with either DS or heparin to provide sustained delivery of BMP-2 and compared to HA/BMP-2 without DS or heparin (Fig. 5). *In vitro* release studies revealed that HA hydrogels with either DS or heparin had an extended release of BMP-2 compared to HA-only hydrogels. Both DS and heparin provided sustained release over 30 days, but DS promoted slightly higher release in the first day. These results are consistent with a binding analysis, showing that heparin had 10 times higher affinity for BMP-2 than DS. HA hydrogels complexed with either DS or heparin stimulated twice the bone formation of the HA hydrogel alone [51]. The lower affinity of DS for BMP-2 may seem surprising when considering DS as a CS subtype; however, DS is structurally different, possessing a 2-O sulfated iduronic acid residue instead of a sulfated glucuronic acid, a structure which is also present in heparin. These studies suggest that binding interactions with BMP-2 are dominated by the sulfation pattern rather than degree of sulfation, and that the presence of glucuronic acid enhances that interaction.

HA-based biomaterials for delivery of BMPs often show poor retention efficiency, and therefore are often combined with other sulfated GAGs. For example, a study showed that the presence of heparin in HA-hydrogels doubled the loading capacity for BMP-2 and reduced its release to 40% during the first 4 days, prolonging sustained delivery over 28 days [50]. Another study compared the efficacy of Pluronic-127 hydrogels containing either CS and HA for delivery of BMP-2 in cartilage regeneration. Although release studies were made using BSA as model protein, the effects on chondrogenesis mediated by BMP-2 in the presence of CS were more pronounced than with HA, while the latter was more efficient than Pluronic hydrogels alone [52]. HA-heparin microgels were fabricated to improve sustained release of BMP-2, using an inverse emulsion polymerization technique, with a loading efficiency of ≈ 90%. No significant burst phase was observed, with 60% release observed over a period of 15 days [53]. These results suggest that heparin and CS are the main GAGs involved in BMP-2 sequestration, while HA does not seem to have strong binding affinity but is useful as a copolymer due to its mechanical properties and biocompatibility. The degree of HA sulfation was studied in an attempt to improve binding efficiency of HA for BMP-4. Use of HA with the highest sulfation degree led to the strongest interaction, followed by HA with low sulfation. Unmodified HA, HS and monosulfated CS (CS-A, CS-C) showed significantly lower binding affinity [157].

Marine GAGs are also likely to interact with TGF-β1, given their structural similarities to other TGF-β1-interacting mammalian GAGs. Carrageenan-based hydrogels were used to encapsulate both TGF-β1 and adipose-derived stem cells (ADSCs) [24]. Although no studies on loading and release of the growth factor were performed, the presence of TGF-β1 in the hydrogels led to an increased expression of chondrogenic markers on the encapsulated ADSCs after 14 days, compared to controls. Another study showed that fucoidan was capable of interacting with and modulating the activity of TGF-β1, promoting proliferation and wound repopulation in an *in vitro* model of wound healing, similar to heparin controls [25].

To conclude, there are many possible GAG/BMP combinations, some of which have yet to be investigated. So far, there appears to be a consensus that CS possesses higher affinity for BMP-2 and BMP-4 than heparin, and therefore would be the most suitable candidate to provide extended release, while heparin would provide release over shorter timescales. In terms of biological activity, release of BMP-4 from CS-E sulfate has been shown to enhance bone formation as compared to heparin, as previously mentioned [156]. Overall, these studies suggest that interactions between GAGs and BMP-2, BMP-4, and BMP-7 are driven not only by bulk electrostatic interactions but are also affected by the location-specific presence of specific monosaccharides and sulfation patterns of the disaccharide units forming the GAG. This is illustrated by the low affinity of BMP proteins for HA, which is negatively charged (although weakly) but lacks sulfate groups.

### 2.7 Neural growth factors

GAGs have multiple, apparently antagonistic roles in regulation of neuronal growth, axon guidance and plasticity in the nervous system [158]. GAGs promote neural growth by binding and presenting growth factors such neural growth factor (NGF), human glial cell line-derived neurotrophic factor (GDNF), pleiotrophin (PTN), midkine (MK) and brain-derived neurotrophic factor (BDNF) to stem and neuronal cells. However, GAGs can also inhibit growth and limit plasticity of neurons when interacting with transmembrane receptors such as tyrosine phosphatase σ (PTPσ), Nogo (NgR1 and NgR3) and leukocyte common antigen related receptors (LAR) [[158], [159], [160]]. For regeneration of neural tissue, there may therefore be specific GAGs that would be highly appropriate as a growth factor carrier, in that they may enhance signaling – and others that might be disadvantageous, inhibiting regeneration. The native biological role of the GAG is likely to be important to the selection process. Secondary modifications to the GAGs themselves may be equally as important as the primary structure.

Heparin has again been used to delivery neural growth factors. A fibrin-heparin scaffold was made to release NGF for sciatic nerve regeneration, which resulted in an increase of neural tissue, as well as higher density and diameter of nerve fibers, suggesting the presence of more mature regenerating nerves [78]. However, CS and DS are major components of the central nervous system, and orchestrate neural progenitor activity through a variety of sulfation patterns in their constitutive sugar units [161]. CS and DS have been investigated as vehicles for delivery of neural growth factors. While binding studies have shown that MK and PTN have high affinity for both disulfated CS-D and CS-E, monosulfated CS-A and CS-C did not show strong binding affinity [[162], [163], [164]]. Similarly, NGF, BDNF and other neurotrophins were found to preferentially bind CS-E rather than CS-A or CS-C [165,166]. Similarly, DS and HS can bind BDNF, MK, and PTN to promote neural growth [10,[167], [168], [169]]. Binding studies showed that the affinity of GDNF for heparin and HS is mostly dependent on the 2-O-sulfation of the iduronic acid residue [160].

**Fig. 6:**
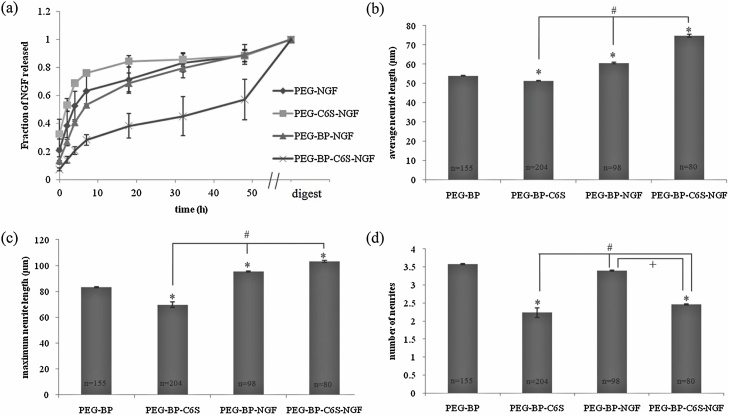
(a) NGF release profile of gels with and without BP and C6S. NGF release was monitored over 48 h. After 2 d, the gels were digested and the amount of NGF quantified. Points represent the mean ± SEM. Effect of C6S and NGF on cortical neuron outgrowth. Neurons were cultured on gels with and without C6S and/or NGF for 48 h. The average neurite length (b), maximum neurite length (c), and number of neurites (d) were determined for each gel composition. Bars represent the mean ± SEM, * p < 0.05 relative to PEG-BP, # p < 0.05 relative to PEG-BP-C6S, + p < 0.05 relative to PEG-BP-NGF. Adapted from [54].

A CS-based hydrogel was developed for the release of NGF, the rate of which was directly correlated to the rate of enzymatic degradation of the hydrogel, releasing its content over 48 h (Fig. 6). Interestingly, the implantation of gels containing solely 6O-sulfated CS-C resulted in inhibition of cortical outgrowth, which was partially overcome by the released NGF [54]. In this instance, a GAG was chosen that was arguably antagonistic to the intended effect, emphasizing that binding affinity is not the only factor that should be considered when choosing a GAG for protein delivery. Similarly, a comparative study of GAG-based hydrogels for the release of NGF showed that the binding affinity of CS is ten-fold stronger that heparin, but lower than HA. Regardless, CS was shown to be a better biomaterial for the hydrogel due to a more robust neurite growth in culture compared to HA-based hydrogels, which suggested that HA may exert inhibitory effects [55]. Despite the potential biological advantages of GAGs for the delivery of neural growth factors, the development of such systems has been relatively limited. These GAGs are less readily available, their *in vivo* roles and interactions less well-understood, and regeneration of neural tissue is often experimentally challenging to achieve.

## 3 GAG-mediated delivery of cytokines and chemokines

As with growth factors, GAGs alone or as proteoglycans bind cytokines and chemokines to regulate cell recruitment, inflammation and tissue remodeling. Native GAGs in the ECM act as a reservoir for pro-inflammatory cytokines such as interleukin (IL)-1β, IL-6, IL-8, IL-10, and tumor necrosis factor (TNF)-α, which were released by overexpression of heparanase [80]. The activity of IL-10 is known to be modulated by HS, heparin and CS during inflammatory processes, influencing Th2 and Th1 immune responses [170,171], and KS proteoglycans have key roles in the control of inflammatory signaling in bone, cartilage and the cornea [172]. Although the use of GAG-based biomaterials for delivery of these molecules has not been fully explored, a wider diversity of GAGs has been studied and used for delivery, serving as a useful example of the advantageous properties of alternative GAGs for protein delivery. In general, binding studies on GAGs suggest that the affinity for some interleukins (*e.g.* IL-10, IL-12), is mainly mediated by relatively unspecific electrostatic interactions between positive charge-rich domains and the negatively-charged sulfate and carboxylic acid residues of GAGs [173,174]. For example, the GAG-binding site of IL-10 is made of several positively charged residues, and has been shown to bind heparin, CS, and HA, as well as DS to a slightly lesser extent [170,173]. In the particular case of IL-6, interactions with GAGs appear to be more specific, as it binds to heparin and fucoidan but not to other GAGs with similar degree of sulfation such as DS and HS, or to monosulfated GAGs such as CS-A and CS-C [175]. In fact, fucoidan showed higher potency, as a similar binding efficiency was obtained with a lower dose [175]. These interactions are partially driven by 2-O, 6-O and N- sulfation in the sugar residues of heparin, although the 2-O sulfation was shown to be non-essential for binding [175]. Other interleukins have shown preferential binding for heparin and HS, including subtypes 2, 3, 4, 5, 7, 8 and 12, in all cases mediated by similar interactions [[176], [177], [178], [179], [180], [181], [182]]. With all interleukins with which fucoidan was tested and compared to heparin (IL-2 and IL-6), fucoidan has shown either similar or superior binding [175,176]. Fucoidan therefore represents a promising animal-free alternative to heparin as a GAG-based biomaterial for delivery of interleukins, although its performance as a delivery system for cytokines has not been studied yet. Similarly, interactions with human transforming growth factor - β1 (TGF-β1) are mediated by charge, but in this case only in GAGs with sulfate groups at two or more disaccharide residues, which include heparin, DS and CS-E. HA is only capable of binding TGF-β1 when sulfated derivatives are made [183].

Heparin has been exploited as a biomaterial for cytokine delivery in the form of hydrogels [57,117], other scaffolds [77], nanoparticles [64], and coacervates [70,72,73]. Heparin-based hydrogels have been made to provide sustained delivery of IL-4 for a period of more than two weeks, leading to more effective polarization of macrophages towards a regulatory (M2) phenotype than IL-4 alone [117]. Similarly, heparinized PCL elastomers functionalized with IL-4 provided sustained release over 7 days and promoted an M2 macrophage phenotype [77]. Stromal cell-derived factor 1 alpha (SDF-1α, also known as CXCL12), a chemokine that elicits recruitment of lymphocytes and hematopoietic stem cells [[184], [185], [186]], has shown to be efficiently loaded into heparin-based hydrogels in the range of 2.5–15 μg/mL, providing a burst release during the first 6 h followed by sustained release over a period of one week. Sustained delivery of SDF-1α resulted in early infiltration of endothelial progenitors and improved vascularization after subcutaneous implantation in a mouse model [57]. SDF-1α has also been loaded into self-assembled heparin/chitosan-oligosaccharide nanoparticles in combination with VEGF. The sustained delivery of SDF-1α and VEGF promoted migration and proliferation of endothelial cells respectively [64]. The affinity of heparin for chemokines can also be used to remove cytokines rather than deliver them. A heparin-based hydrogel was used as a wound-dressing to improve wound healing in mice, by scavenging and sequestering inflammatory chemokines from the wound. These included monocyte chemoattractant protein-1 (MCP-1), IL-8, macrophage inflammatory protein-1α (MIP-1α) and MIP-1β [187].

Heparin/PEAD coacervates have been shown to be a simple and versatile GAG-based delivery system to efficiently load and deliver single and multiple therapeutic proteins. The fabrication of coacervates involves electrostatic interactions between heparin and PEAD rather than crosslinking or modification of heparin, leaving its binding sites for growth factors and cytokines intact. Heparin coacervates for delivery of IL-10 and Sonic Hedgehog (SHH) have been co-injected within a degradable hydrogel into rat hearts after myocardial infarction (MI). Results after four weeks post-MI revealed that scar reduction and myocardial vascularization were synergistically improved compared to single delivery, and that the combined regimen was critical to preserving heart function [72]. Similarly, these coacervates were capable of delivering both IL-10 and FGF-1 in a spatially and temporally-controlled manner and showed anti-inflammatory effects and improvements in infarct size, revascularization and heart contractility [70]. Coacervates have also been used to simultaneously deliver VEGF, MCP-1 and IL-6, with maximum loading efficiencies obtained when these proteins are included at a ratio of 25:5:1, respectively. No interference in release was observed between the incorporated proteins; with a release of 19%, 29% and 18% after 24 h for VEGF, MCP-1 and IL-6, respectively. Delivered VEGF stimulated endothelial cell proliferation, MCP-1 increased macrophage migration and IL-6 stimulated IgM production *in vitro* [73].

The anti-inflammatory activity of CS is widely recognized and is thought to act mostly *via* the inhibition of NF-κB nuclear translocation [92,[188], [189], [190]]. Surprisingly, DS, predominantly found during inflammatory phases of wound healing, was found to rapidly activate NF-κB translocation after injury [191]. These reported differences are likely due to the use of CS mixtures rather than isolated subtypes, while other studies have specifically confirmed NF-κB inhibition in the presence of CS-A (4-O sulfate) and CS-C (6-O sulfate) [189,192]. Regardless, the anti-inflammatory effects of both CS and DS have been also shown to be mediated by binding and regulation of cytokines and chemokines, which are dependent not only on sulfation degree but also sulfation pattern and the conformation of their sugar units [193]. CS subtypes A through E have shown distinct affinities for numerous cytokines, as a consequence of their multiple sulfation degrees and patterns [193,194]. As before, their native biological roles in wound healing are potentially advantageous to their use as drug delivery vehicles but should be considered in the context of the injury type and the cytokines to be delivered in order to avoid antagonistic effects.

CS, DS and its derivatives have been used as biomaterials for the delivery of cytokines in particle-based systems, micelles, and eluting-coatings [61,68,118]. For example, CS microspheres were shown to strongly sequester positively charged TGF-β1 (mediated by electrostatic interactions with CS), releasing a minimal amount by diffusion. Contrastingly, negatively charged TNF-α was mostly released during the first 15 h [68]. Although it was not tested, it is likely that release of TNF-α would be even faster when enzymatic degradation occurs (*e.g.* using chondroitinases or *in vivo*), while TGF-β1 would be released in a sustained manner.

**Fig. 7:**
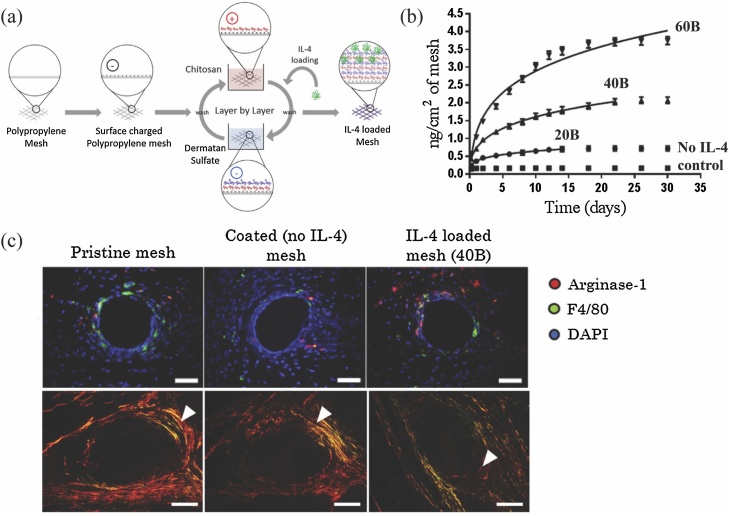
(a) Multilayered Chitosan/DS-coated implant for sustained delivery of IL-4. (b) Cumulative release profiles of coatings containing 20, 40 and 60 bilayers of IL-4. (c) Fluorescence microscopy images of (a) Arginase-1 (red, M2 macrophage marker) and F4/80 (green, pan-macrophage marker) co-immunolabeled tissue sections, 7 days post-implantation (top panel). Scale bars: 50 μm. Picro Sirius Red stained tissue sections, 90 days post-implantation (bottom panel). Arrowheads indicate the fibrotic capsule surrounding single implant fibers. Scale bars represent 100 μm. Adapted from [118] and reprinted with permission from Elsevier (For interpretation of the references to colour in this figure legend, the reader is referred to the web version of this article).

Even though DS is also sometimes considered a CS subtype, it has distinct structural differences with functional consequences. In fact, DS shares the same 2-O sulfated iduronic acid sugar of heparin/HS and N-acetyl galactosamine of CS, which gives DS a remarkable binding specificity for a subset of growth factors and cytokines, especially those with anti-inflammatory activity [194,195]. DS has been used as a polyelectrolyte biomaterial in multilayered eluting-coatings for the loading and release of cytokines for immunomodulation of the host response against biomaterials (Fig. 7) [61,118]. These coatings showed that both the amount and length of release of cytokines can be tuned according to the number of bilayers forming the coating, providing a sustained release of a few nanograms of cytokine over a period ranging from a few days to weeks. Sustained release of IL-4 from polypropylene implants over approximately 2 weeks shifted the inflammatory (M1) response of macrophages towards a regulatory (M2)-like response, a shift associated with reduced scarring and improved integration of the implant into the host tissue [118]. DS was chosen over other GAGs in this study as DS was found to bind and enhance the activity of IL-4 *in vivo*, while other GAGs showed no binding (*e.g.* HS, CS-A, CS-C, CS-D and CS-E) or bound without enhancing signaling (*e.g.* heparin) [194,195]. Due to the multilayered nature of these coatings, cytokines can be also released in a sequential manner. A follow-up study released MCP-1 and IL-4 in a sequential manner to restore macrophage recruitment and promote an M2-like macrophage response in aged animals [61]. External multilayers containing MCP-1 were able to release 80% of MCP-1 during the first week, while the internal multilayers containing IL-4 provided sustained release up to 16 days [61]. It is worth mentioning that the fabrication of the coating is mediated by electrostatic interactions between the two oppositely charged polyelectrolytes (DS and chitosan in this case), and hence no chemical modification of DS was performed, leaving all sites intact for interaction with cytokines.

As HA can sequester cytokines and chemokines through interactions mediated by non-sulfated sugar residues, it has also been used as a biomaterial to provide sustained release in the form of hydrogels [56,58] and multilayered films [119]. Injectable hydrogels made of adamantane-modified HA and cyclodextrin were developed to provide sustained delivery of IL-10, the release of which was correlated with the rate of hydrogel degradation, resulting in reduced systemic inflammation and improved renal function in a mouse acute kidney injury model [56]. Sustained delivery of both SDF-1α and BMP-2 was obtained from MMP-degradable HA-based hydrogels, the release ratio of which was again dependent on degradation (*via* enzymatic activity) and provided up to 10 days of release with a minimal burst release [58]. HA was also used as a polyanion in combination with poly-lysine (PLL) to fabricate multilayered films for the release of SDF-1α. The amount of chemokine loaded in these films ranged from 100 ng/cm^2^ to 5 mg/cm^2^, depending on SDF-1α concentration, pH and degree of crosslinking. Released SDF-1α increased myoblast migration, spreading and myogenic marker expression [119].

## 4 Future directions and concluding remarks

Glycosaminoglycans are increasingly attracting attention in the medical field and are becoming part of a new generation of biomimetic biomaterials, due to their exceptional biocompatibility, tunability, biological effects, and mechanical properties. Heparin-based biomaterials have been shown to be generally effective for a variety of applications, and for certain proteins and biological contexts are indeed the most appropriate choice. However, to reach the full potential of GAG-based biomaterials, the field should aim to transition beyond the use of heparin towards more rational and specific choices. Specific focus on more biologically relevant GAG candidates able to mimic the biological activity and bioavailability of growth factors and cytokines in the target tissue could allow the achievement of more predictable and appropriate therapeutic effects. Therefore, a better understanding of the nature and biological relevance of the interactions between GAGs and proteins is of paramount importance. Experimental and modelling techniques are becoming increasingly competent to perform more appropriate characterization of these interactions, but additional advances will likely be required to fully understand these binding mechanisms, which vary from highly promiscuous to extremely specific. When previous studies on a particular GAG-protein interaction are not available to make a rational choice of a GAG candidate for delivery purposes, preliminary studies to compare binding affinities (*e.g.* using commercial GAG microarrays or surface plasmon resonance) are recommended. Increased integration of GAGs within complex, multifunctional semi-synthetic materials is anticipated as a future development – this will require the development of chemically-modified GAG derivatives with novel functional groups. Thus, the impacts of these modifications on the binding affinity and biological performance of these materials will need to be considered accordingly. Understanding specific GAG-protein interactions will also allow identification of the residues in the disaccharide units which are essential for interaction. This will allow prediction of chemical modifications that would be compatible with the preservation of these interactions, and which modifications may alter them and to what extent. Besides their ability to act *via* release of exogenously loaded therapeutic proteins, GAGs themselves can act as therapeutic agents by their interactions with proteins already present in the body. For example, heparin is a powerful anticoagulant, and a heparin-containing hydrogel capable of regulating blood coagulation has been developed [196,197]. This hydrogel was able to release heparin upon the enzymatic cleavage of crosslinking groups, providing a protective anticoagulant activity. KS has shown therapeutic potential in inflammatory arthritis by suppressing cartilage damage and inflammation [198]. Use of a therapeutically active GAG in addition to an exogenous therapeutic can achieve a synergistic effect; therefore, the biological functions of GAGs should be also considered when choosing candidates for specific applications. As with many other materials of natural origin, GAG-based biomaterials face challenges in clinical and industrial translation associated with the complex standardization procedures necessary due to the high potential for variability of materials from biological sources. Finally, improvements in scaling and isolation methods are required to obtain other, less commonly-used but otherwise advantageous GAGs at a scale and cost that is suitable for biomaterial fabrication, as currently some of these materials (*e.g.* some pure CS subtypes, HS and KS) can only be obtained at the milligram-scale at high cost. Marine-derived GAG biomaterials represent promising animal-free biomaterial alternatives that can be scaled-up relatively inexpensively for biomaterial fabrication in a biomedical industry setting.

GAGs represent a promising class of natural materials able to sequester and release a variety of protein cargoes for diverse therapeutic applications. However, although many GAGs are able to sequester many proteins in a non-specific manner, GAGs are not all alike. Some may be more or less suitable for specific applications as a result of their biological signaling properties, interactions with cargo, degradability, adaptability for modifications, commercial availability and immunological offensiveness. These properties are not yet fully described or understood for all GAGs. As these properties continue to be described in greater detail for less commonly-used GAGs, their utility for therapeutic applications will continue to emerge, allowing their exploitation in the next generation of protein drug delivery systems.
