# Supplementary material for: Glycosaminoglycan-based biomaterials for growth factor and cytokine delivery: Making the right choices
Source: J Control Release. 2019 Nov 10;313:131–47. doi: 10.1016/j.jconrel.2019.10.018 (PMC6900262; doi:10.1016/j.jconrel.2019.10.018)
Supplement: Supplementary file 1 [file mmc1.pdf]

Proceedings of the
National Academy of Sciences
of the United States of America

NEW RESEARCH IN

Physical Sciences

Social Sciences

Biological Sciences

Rights and Permissions

← About

The author(s) retains copyright to individual PNAS articles, and the National Academy of Sciences of the United States of America (NAS) holds copyright to the collective work and retains an exclusive License to Publish these articles, except for open access articles submitted beginning September 2017. For such open access articles, NAS retains a nonexclusive License to Publish, and these articles are distributed under either a CC BY-NC-ND or CC BY license.

For volumes 106–114 (2009–September 2017), the author(s) retains copyright to individual articles, and NAS retains an exclusive License to Publish these articles and holds copyright to the collective work. Volumes 90–105 (1993–2008) are copyright National Academy of Sciences. For volumes 1–89 (1915–1992), the author(s) retains copyright to individual articles, and NAS holds copyright to the collective work.

Authors whose work will be reused should be notified. Use of PNAS material must not imply any endorsement by PNAS or NAS. The full journal reference must be cited and, for articles published in Volumes 90–105 (1993–2008), "Copyright (copyright year) National Academy of Sciences" must be included as a copyright note.

Please visit the Permissions FAQ for detailed information about PNAS copyright and self-archiving guidelines. The PNAS listing on the Sherpa RoMEO publisher policies pages can be found here.

Additional information and answers to frequently asked questions about author rights and permissions are available on our FAQ page.

PNAS authors need not obtain permission for the following cases:

1. to use their original figures or tables in their future works;
2. to make copies of their articles for their own personal use, including classroom use, or for the personal use of colleagues, provided those copies are not for sale and are not distributed in a systematic way;
3. to include their articles as part of their dissertations; or
4. to use all or part of their articles in printed compilations of their own works.

The full journal reference must be cited and, for articles published in volumes 90–105 (1993–2008), "Copyright (copyright year) National Academy of Sciences" must be included as a copyright note.

Requesting Permission

For permission to reuse material in volumes 1–89 (1915–1992), requests should be sent to the original authors, who hold the copyright. The full journal reference must be cited.

For permission to reuse material in volumes 90–114 (1993–2017), requests should be sent to PNASpermissions@nas.edu and must include the following information about the requestor and the material:

1. Your full name, affiliation, and title
2. Your complete mailing address, phone number, and email
3. PNAS volume number, issue number, and issue date
4. PNAS article title
5. PNAS authors' names
6. Page numbers of items to be reprinted
7. Figure/table number or portion of text to be reprinted

Requests must also include the following information about the intended use of the material:

1. Title of work in which PNAS material will appear
2. Authors/editors of work
3. Publisher of work
4. Retail price of work
5. Number of copies of work to be produced
6. Intended audience
7. Whether work is for nonprofit or commercial use

Permission is not required to use original figures or tables for noncommercial and educational use (i.e., in a review article, in a book that is not for sale) if the article published under the exclusive PNAS License to Publish. Please include a full journal reference and, for articles published in volumes 90–105 (1993–2008), include "Copyright (copyright year) National Academy of Sciences" as a copyright note. Commercial reuse of figures and tables (i.e., in promotional materials, in a textbook for sale) requires permission from PNAS.

Reproducing full articles (whether physically or electronically) published under the exclusive PNAS License to Publish requires permission from PNAS and will also incur an article reprint fee. Sharing the URL of a PNAS article does not require permission and will not incur any fees.

For permission to reprint material in volumes 114–present (2017–present), requests relating to articles published under the exclusive PNAS License to Publish should be sent to PNASpermissions@nas.edu. For open access articles that are distributed under the CC BY-NC-ND or CC BY license, permission may not be required. Users are advised to check each article for its publication license and corresponding reuse and distribution policies.

Text and data mining are permitted for noncommercial institutions with an active institutional site license to PNAS for internal noncommercial research purposes. Other requests should be sent to PNASpermissions@nas.edu.

PNAS cannot supply original artwork. Such requests should be directed to the article's corresponding author (contact information available in the article's footnotes).

Additional Permissions Requests

Copyright Clearance Center offers the following pay-per-use options for PNAS material:

- Use in electronic or print course materials (i.e. coursepacks, classroom handouts, posting on an academic institution intranet)
- Delivery via Interlibrary Loan (ILL) or other document delivery
- Photocopying for general business or academic use
- Sharing content electronically (i.e. sending in an e-mail, posting on an intranet or extranet)

Pay-per-use orders can be submitted directly through the Copyright Clearance Center website.

Authorization to photocopy items for the internal or personal use of specific clients is granted by the NAS provided that the proper fee is paid directly to the Copyright Clearance Center.

For questions regarding any of the above, including permission to photocopy beyond that permitted by Section 107 or 108 of the US Copyright Law, contact:

Copyright Clearance Center 222 Rosewood Drive Danvers, MA 01923 Phone: 978-750-8400 Fax: 978-750-4770 Email: info@copyright.com

[07/19]

Submit Manuscript

Twitter

Facebook

RSS Feeds

Email Alerts

Articles

Current Issue

Latest Articles

Archive

PNAS Portals

Classics

Front Matter

Teaching Resources

Anthropology

Chemistry

Physics

Sustainability Science

Information

Authors

Editorial Board

Reviewers

Press

Site Map

PNAS Updates

Feedback Privacy/Legal

Copyright © 2019 National Academy of Sciences. Online ISSN 1091-6490
