# Supplementary material for: Glycosaminoglycan-based biomaterials for growth factor and cytokine delivery: Making the right choices
Source: J Control Release. 2019 Nov 10;313:131–47. doi: 10.1016/j.jconrel.2019.10.018 (PMC6900262; doi:10.1016/j.jconrel.2019.10.018)
Supplement: Supplementary file 3 [file mmc3.pdf]

PLOS | ONE

Complexation and Sequestration of BMP-2 from an ECM Mimetic Hyaluronan Gel for Improved Bone Formation

Marta Kisiel, Agnieszka S. Klar, Manuela Ventura, Jos Buijs, Marc-Krystelle Mafina, Simon M. Cool, Jöns Hilborn

Published: October 22, 2013 • https://doi.org/10.1371/journal.pone.0078551

Abstract

Bone morphogenetic protein-2 (BMP-2) is considered a promising adjuvant for the treatment of skeletal non-union and spinal fusion. However, BMP-2 delivery in a conventional collagen scaffold necessitates a high dose to achieve an efficacious outcome. To lower its effective dose, we precomplexed BMP-2 with the glycosaminoglycans (GAGs) dermatan sulfate (DS) or heparin (HP), prior to loading it into a hyaluronic acid (HA) hydrogel. *In vitro* release studies showed that BMP-2 precomplexed with DS or HP had a prolonged delivery compared to without GAG. BMP-2-DS complexes achieved a slightly faster release in the first 24 h than HP; however, both delivered BMP-2 for an equal duration. Analysis of the kinetic interaction between BMP-2 and DS or HP showed that HP had approximately 10 times higher affinity for BMP-2 than DS, yet it equally stabilized the protein, as determined by alkaline phosphatase activity. Ectopic bone formation assays at subcutaneous sites in rats demonstrated that HA hydrogel-delivered BMP-2 precomplexed with GAG induced twice the volume of bone compared with BMP-2 delivered uncomplexed to GAG.

Citation: Kisiel M, Klar AS, Ventura M, Buijs J, Mafina M-K, Cool SM, et al. (2013) Complexation and Sequestration of BMP-2 from an ECM Mimetic Hyaluronan Gel for Improved Bone Formation. PLoS ONE 8(10): e78551. https://doi.org/10.1371/journal.pone.0078551

Editor: Nikos K Karamanos, University of Patras, Greece

Received: June 9, 2013; **Accepted:** September 20, 2013; **Published:** October 22, 2013

Copyright: © 2013 Kisiel et al. This is an open-access article distributed under the terms of the Creative Commons Attribution License, which permits unrestricted use, distribution, and reproduction in any medium, provided the original author and source are credited.

Funding: This work was supported by the European Community's Seventh Framework Programme (MultiTERM, grant agreement No. 238551 and BIODESIGN, grant agreement No. 262948). The funders had no role in study design, data collection and analysis, decision to publish, or preparation of the manuscript.

Competing interests: Jos Buijs, who worked for Science for Life Laboratory, GE Healthcare, Stockholm and currently for Biomedical Radiation Sciences, Uppsala University declares no competing interests. This does not alter the authors' adherence to all the PLOS ONE policies on sharing data and materials.

Introduction

Worldwide, patients continue to suffer from bone non-unions. Gold standard treatment relies on the continued use of autologous bone graft obtained from the patient's own iliac crest [1]. This bone source has a limited quantity and the quality is dependent on the individual patient, which reduces its therapeutic potential [2]. Thus, bone repair by tissue engineering systems has attracted broad attention. Despite the continuing development of hormones and other bone-stimulating molecules, bone morphogenetic proteins (BMPs) remain the most potent inducers of bone formation *in vivo* [3]. In particular, BMP-2 is widely recognized to be one of the most powerful osteoinductive factors for bone regeneration [4,5] and was originally identified as a factor in bone tissue that in extracted form could stimulate bone formation when added exogenously to an extraosseous site [6]. Moreover, human recombinant BMP-2 [7], has proven to be highly efficient as a bone-inducing adjuvant in animals.

Endogenous BMP-2 is also important for normal bone homeostasis and is upregulated immediately following bone trauma [8] and actively contributes to the recruitment, proliferation and differentiation of osteoprogenitor cells during the bone healing process [9]. In the clinical setting, BMP-2 absorbed into a bovine collagen type I sponge has proven to be effective in the treatment of degenerative disc disease (spinal fusion) and fracture non-union [10,11]. However, excessive dosing has been associated with adverse events that include tissue edema and ossification at undesired sites [12,13]. There is also concern because the systemic half-life of BMP-2 is short and FDA-approved delivery is reliant on a collagen sponge with low affinity for BMP-2 [14], so requiring supra-physiological doses in order to achieve an efficacious outcome [15].

Recent evidence by our group and others [16,17] suggests that BMP-2-induced bone formation is largely dependent on stability of BMP-2 and its release kinetics, with a controlled release enhancing the effect. Long-term BMP-2 delivery increases bone-healing rates compared with short-term delivery at an equal dose [18,19]. As a consequence, a number of delivery strategies aimed at improving BMP-2 dose-effectiveness have been developed. Our group, along with others, has shown that hyaluronic acid (HA) hydrogels are suitable for bone tissue engineering applications [20-23]. HA is a natural extracellular matrix glycosaminoglycan (GAG) that regulates several biological processes, including cell migration, proliferation, differentiation and wound healing [24]. *In vivo*, HA is degraded by the action of oxygen free radicals [25] and hyaluronidases [26,27]. Also, HA hydrogels are non-immunogenic and have been successfully utilized as scaffolds for BMP-2 delivery both in preclinical [20,28,29] and clinical [13] trials. As an injectable device, HA permits *in situ* administration in a minimally invasive manner [21,30]. Although promising characteristics, HA hydrogels share a problem with many similar materials, namely insufficient control of BMP-2 release. This is because many hydrogels rapidly releases BMP-2 through a passive diffusion mechanism [28]. Although BMP-2 could be covalently linked to this polymeric scaffold [31] such a chemical modification may compromise BMP-2 activity. Also, electrostatic immobilization of BMP-2 on a basement membrane proteoglycan (perlecan domain I) covalently conjugated to a HA hydrogel has been attempted [32]. However this strategy whilst sustaining the release of active BMP-2, is limited by the elaborated multi-step bioconjugation.
