# Supplementary material for: Glycosaminoglycan-based biomaterials for growth factor and cytokine delivery: Making the right choices
Source: J Control Release. 2019 Nov 10;313:131–47. doi: 10.1016/j.jconrel.2019.10.018 (PMC6900262; doi:10.1016/j.jconrel.2019.10.018)
Supplement: Supplementary file 4 [file mmc4.pdf]

Taylor & Francis Online

Log in | Register

Journal

**Biomatter** ›

Volume 1, 2011 - Issue 2

Open access

243 Views | 12 CrossRef citations to date | 1 Altmetric

Report

# Development of affinity-based delivery of NGF from a chondroitin sulfate biomaterial

Karen Chao Butterfield, Aaron W. Conovaloff & **Alyssa Panitch**

Pages 174-181 | Received 21 Jul 2011, Accepted 16 Nov 2011, Published online: 01 Oct 2011

Download citation https://doi.org/10.4161/biom.18791

Full Article Figures & data References Citations Metrics

Licensing PDF

Copyright © Landes Bioscience

This is an open-access article licensed under a Creative Commons Attribution-NonCommercial 3.0 Unported License. The article may be redistributed, reproduced, and reused for non-commercial purposes, provided the original source is properly cited.

## People also read

Brief report
